# Frostbite and Cold Agglutinin Disease: Coexistence of Two Entities Leading to Poor Clinical Outcomes

**DOI:** 10.3390/medicina57060592

**Published:** 2021-06-08

**Authors:** Noel Lorenzo-Villalba, Emmanuel Andres, Javier Guerrero-Niño, Edward Nasco, Jessy Cattelan, Xavier Jannot, Marie-Pierre Ledoux

**Affiliations:** 1 Service de Médecine Interne, Diabète et Maladies Métaboliques, Hôpitaux Universitaires de Strasbourg, 67000 Strasbourg, France; emmanuel.andres@chru-strasbourg.fr (E.A.); edward.nasco@chru-strasbourg.fr (E.N.); jcattelan@hotmail.fr (J.C.); xavier.jannot@chru-strasbourg.fr (X.J.); 2 Service des Urgences, Hôpitaux Universitaires de Strasbourg, 67000 Strasbourg, France; javier.guerrero@chru-strasbourg.fr; 3 Service d’Hématologie, Hôpitaux Universitaires de Strasbourg, 67000 Strasbourg, France; marie-pierre.ledoux@chru-strasbourg.fr

**Keywords:** frostbite, cold agglutinin disease, amputation

## Abstract

An 83-year-old woman was admitted to the emergency department for a 7-day history of fatigue and progressive cyanosis in the feet and hands after cold exposure despite physical protective measures. Upon arrival, the patient presented with necrotic cutaneous lesions in both hands and distal lower extremities. Upon admission, hemoglobin was 7.6 g/dL and laboratory tests were consistent with cold agglutinin disease (CAD), the presence of monoclonal IgM, and flow cytometry consistent with lymphoplasmacytic lymphoma, but MYD88 L265P mutation was negative. The patient required blood transfusion, resulting in stabilized hemoglobin and a decrease in markers of hemolysis. Treatment with aspirin 250 mg daily and intravenous iloprost 0.5 mL/h was initiated with a poor clinical response at day 4. Amputation was required. Plasma exchange was performed and chemotherapy with rituximab and bendamustine was initiated. The clinical course was marked by further necrosis, prompting discussions regarding an additional amputation that was not performed considering the high surgical risk and refusal by the patient. Supportive treatment was initiated, and the patient expired one month after hospital admission.

## 1. Introduction

Cold agglutinin disease (CAD) is a rare clonal B cell lymphoproliferative disorder, and its prevalence in Northern Europe has been estimated to be 16 cases per million inhabitants and the incidence rate to be 1 per million per year [1]. CAD accounts for 15–25% of cases of autoimmune hemolytic anemia with a median age of about 76 years [1]. CAD must be distinguished from secondary cold agglutinin syndrome (CAS) arising in the setting of specific infections or malignancy such as aggressive lymphoma. The involved autoantibodies, known as cold agglutinins (CAs), bind to their antigen at an optimum temperature of 3–4 °C, but are also able to react at a higher temperature, depending on the thermal amplitude [1]. In contrast, frostbite, also known as freezing cold injury, is tissue damage resulting from cold exposure, occurring at temperatures below 0 degrees C [2]. The coexistence of both conditions could have deleterious functional effects in affected patients.

## 2. Case Presentation

An 83-year-old woman was admitted to the internal medicine department for a 7-day history of fatigue along with progressive cyanosis in the feet and hands despite physical protective measures. Necrotic lesions initially affected both feet, then hands. They developed after exposure to temperatures of about −10 °C for approximately 1 h. The patient reported no improvement after rewarming of the extremities at home.

The patient had no history of fever, chills, or weight loss but reported a progressive shortness of breath during the 48 h prior to hospital admission. Her past medical history was significant for Raynaud’s, atrial fibrillation, and Graves Basedow disease. Medications included bisoprolol 5 mg twice daily, flecainide 100 mg daily, and fluindione 20 mg daily. There were no newly introduced medications. Her family history was noncontributory. She lived alone. She had no known drug or food allergies, as well as no history of smoking, alcohol abuse, or illicit drug use. 

**Figure 1:**
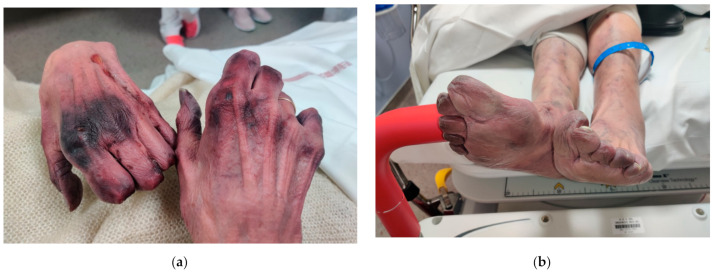
Upon admission at the emergency department (J0). (**a**) Hands, (**b**) Lower limbs.

On physical examination, her heart rate was 130 beats/min, blood pressure was 107/55 mmHg, respiratory rate was 20 breaths/min, and oxygen saturation was 98% on room air. The patient was alert and oriented to time, place, and person. Cyanosis was noted in the feet from the toes to the ankles, as well as in both hands from distal phalanges to the wrist. Necrotic lesions in the phalanges and part of the left metacarpals were noted (Figure 1a,b).

A mild mucocutaneous jaundice was noted. Heart sounds were tachycardic and irregular without any rubs or murmurs. The lungs were clear. The abdomen was tender and nondistended without masses.

Blood tests showed leukocytosis (30 × 10^9^/L) with a predominance of polymorphonuclear neutrophils (24 × 10^9^/L) and monocytosis (2.5 × 10^9^/L) with a normal platelet count, and elevated C-reactive protein (35 mg/L). Hemoglobin (Hb) was 7.6 g/dL, mean corpuscular volume 75 fl with elevated reticulocytes (300 × 10^9^/L). Haptoglobin was undetectable, LDH was 1720 U/L, and both conjugated and non-conjugated bilirubin fractions were increased. No schizocytes were found on peripheral blood examination. Liver function tests showed levels of alanine transaminase (ALT) > 10× normal range and aspartate transaminase (AST) > 3× normal range with normal alkaline phosphatase and gamma-glutamyl transferase. Prothrombin time (PT) was 22% and fibrinogen 1.79 g/L. Creatine kinase and ferritin were also increased, 10,000 U/L and 271 ug/L, respectively. The glomerular filtration rate (GFR) was 66 mL/min, serum creatinine was normal, but serum sodium was 129 mmol/L. The folic acid, vitamin B12 and thyroid function tests were within the normal range. The electrocardiogram showed atrial fibrillation with a rapid ventricular response. The patient was transfused 2 units of blood reaching a hemoglobin level of 9 g/dL.

In this setting of acute hemolysis, a Coombs test was ordered, showing positive direct antiglobulin for anti C3d, negativity for IgG, and a CA titer of ≥64. The diagnosis of CAD was made.

The patient initially received IV fluids, digoxin to control the heart rate, and one blood transfusion. 

After 24 h at the emergency department, she was transferred to the internal medicine unit. The clinical course was marked by a worsening of the cutaneous manifestations, especially in the hands, becoming burgundy colored up to the wrist and sallow colored up the elbow. These necrotic lesions did not occur similarly in the feet, where only the toes showed lesions with a sallow color up to the level of the ankle. Aspirin 250 mg daily as well as intravenous iloprost 0.5 mL/h were initiated. The goal of treatment was to salvage as much tissue as possible so that maximal function remained. Iloprost could not be increased due to the patient’s intolerance (hypotension and headaches). Another 2 units of blood were transfused as hemoglobin dropped from 9 to 7.2 g/dL. The control performed after showed a hemoglobin level of 9.5 g/dL.

Viral serologies (HIV, HBV, HCV, CMV, and EBV) were negative. QuantiFERON was also negative. Antinuclear, antiphospholipid, anti-neutrophilic cytoplasmic antibodies, and cryoglobulins were negative. The total complement (22 U/mL) and C3 and C4 fractions were diminished (0.4 g/L and 0.08 g/L, respectively). Adams-13 activity was 64%. The serum protein electrophoresis showed a monoclonal protein IgM kappa. Flow cytometry showed a monotypic B lymphoproliferation kappa (low intensity) CD5+, CD10-, CD23-, FMC7+, CD20+ (high intensity), CD22+ (high intensity), CD79b+ (high intensity), CD148 weak, CD180 weak, CD200 weak, representing approximately 3% of total circulating cells. The Matutes score was 2 (CD5+ Kappa low). CD38 was negative. The immunophenotypic profile was suggestive of a circulating, non-CLL microclone B, specifically a lymphoplasmacytic lymphoma. MYD88 was negative.

After 3 days of treatment, the necrotic lesions spread proximally at the level of frostbite, but the necrosis no longer progressed beyond the extent present upon admission to internal medicine. On day 5 after iloprost and aspirin were initiated, this treatment was discontinued given the poor response to therapy. Bone scintigraphy with single-photon emission computed tomography (SPECT) to evaluate the depth of the injury was not considered.

**Figure 2:**
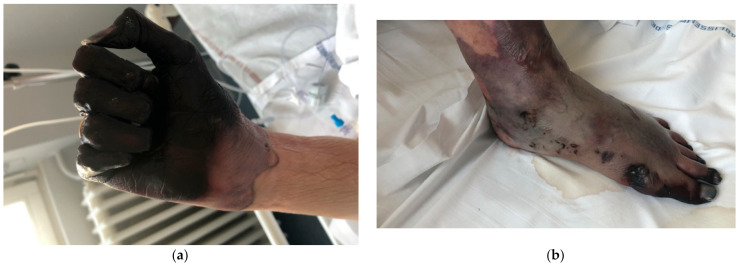
Preoperative images (J7). (**a**) Hands, (**b**) Lower limbs.

The case was discussed with dermatologists, orthopedic surgeons, and vascular surgeons, and the decision was made for amputation (Figure 2a,b).

Plasma exchange to remove the pathological immunoglobulin was performed and chemotherapy with rituximab and bendamustine was initiated. There was frequent bleeding in the stumps requiring the evacuation of hematomas. Necrosis progressed, prompting discussion regarding further surgery. However, the patient refused additional amputation given her high surgical risk. Supportive treatment was initiated, and the patient expired one month after hospital admission.

## 3. Discussion

Patients present with frostbite after exposure to intense cold, resulting in vasoconstriction [2]. The rapid clinical deterioration and the results of the initial laboratory tests at the emergency department revealed the presence of another medical condition which may have also contributed to the poor outcome. As in our patient, the regions that are most commonly affected are the feet and hands.

The median hemoglobin (Hb) level ranged between 8.0 g/dL and 10.4 g/dL, but lower hemoglobin levels as well as compensated hemolysis with a normal Hb are not uncommon [1,3,4]. In the current case, Hb and hemolysis markers remained steady after transfusing two blood units two times. 

In 90% of patients, cold-induced agglutination-mediated symptoms from capillary circulation have been reported, ranging from slight acrocyanosis to disabling Raynaud’s. Less typical and uncommon clinical features include gangrene, livedo reticularis, and livedo racemose [5]. Our patient had previous history of Raynaud’s, but considering the extent and severity of cutaneous manifestations, investigations to rule out the presence of other conditions such as cryoglobulinemia or concomitant cold-reactive IgM were performed. In addition, most patients have an abnormal bone marrow B-cell clone. The concomitant lymphoproliferative disease (LPD) has been most often classified as lymphoplasmacytic lymphoma (LPL)/Waldenström’s macroglobulinemia (WM), followed by marginal zone lymphoma (MZL) and unclassified indolent LPD [4]. Up to 50% of patients diagnosed with primary CAD fulfill the diagnostic criteria for WM, whereas <5% of patients with WM have CAD [6]. Considering the presence of IgM monoclonal protein [1] on immunofixation in our patient, we decided to complete the study with a flow cytometry which was consistent with a lymphoplasmacytic lymphoma.

In one study, bone marrow samples from 54 patients with CAD were systematically re-examined by a group of lymphoma pathologists who noted findings consistent with a homogeneous bone marrow disorder that they termed “primary CAD-associated LPD”. This pattern was different from LPL, MZL, and other previously recognized lymphoma entities [7]. The MYD88 L265P mutation shown to be present in approximately 90% of cases of WM/LPL could not be demonstrated in CAD-associated LPD in our patient [1].

Rituximab monotherapy is the most widely used drug in the treatment armamentarium of CAD. However, combined therapy with bendamustine and rituximab has been shown to be very efficient in primary CAD. The response duration has been reported to be equally long or longer with bendamustine plus rituximab [8]. Treatment with the combination bendamustine–rituximab was associated with lower infection compared to fludarabine–rituximab [8].

## 4. Conclusions

Frostbite and CAD require early identification and treatment to improve outcomes. Regarding the management of CAD patients, early diagnosis of CAD and prevention of CAD attacks such as acrocyanosis and subsequent hemoglobinuria are crucial in avoiding critical complications. The MYD88 L265P mutation is helpful to distinguish between WM/LPL and CAD-associated LPD.
